# Dengue in Pregnancy: A Southeast Asian Perspective

**DOI:** 10.3390/tropicalmed8020086

**Published:** 2023-01-27

**Authors:** Vanessa Chong, Jennifer Zi Ling Tan, Valliammai Jayanthi Thirunavuk Arasoo

**Affiliations:** 1 Monash School of Medicine, Faculty of Medicine, Nursing & Health Sciences, Monash University Australia, Clayton 3168, Australia; 2 Clinical School Johor Bahru, Jeffrey Cheah School of Medicine and Health Sciences, Monash University Malaysia, Johor Bahru 80100, Malaysia

**Keywords:** dengue, pregnancy, Southeast Asia, trimester, physiology, investigations, management

## Abstract

Dengue cases have been rising in recent years. In 2019 alone, over 658,301 of the 5.6 million reported cases originated from Southeast Asia (SEA). Research has also shown detrimental outcomes for pregnant infected women. Despite this, existing literature describing dengue’s effects on pregnancy in SEA is insufficient. Through this narrative review, we sought to describe dengue’s effects on pregnancy systemically and emphasize the existing gaps in the literature. We extensively searched various journals cited in PubMed and Ovid Medline, national clinical practice guidelines, and governmental reports. Dengue in pregnancy increases the risk of pre-eclampsia, Dengue Hemorrhagic Fever (DHF), fetal distress, preterm delivery, Caesarean delivery, and maternal mortality. Vertical transmission, intrauterine growth restriction, and stillbirth are possible sequelae of dengue in fetuses. We found that trimester-specific physiological impacts of dengue in pregnancy (to both mother and child) and investigations and management methods demanded further research, especially in the SEA region.

## 1. Introduction

Dengue is endemic to the Southeastern geographical area of Asia (SEA), spreading through the mosquito vector *Aedes aegypti* [1]. The countries included in the geographical region of SEA include Brunei, Myanmar, Cambodia, Timor-Leste, Indonesia, Laos, Malaysia, Philippines, Singapore, Thailand, and Vietnam [2]. Globally, the incidence of dengue increased from 30,668,000 in 1990 to 56,879,000 in 2019 [3]. In SEA alone, there were 7,700,000 cases of dengue in 2019 [4].

Dengue cases were seen more often in females than males, with the age-standardized incidence rate (ASIR) female-to-male ratio of 1.10 in 1990 and 1.08 in 2019 [3]. In 2019, the ASIR per 100,000 in SEA was 1153.57, the 3rd highest after Oceania and South Asia [4].

The 15–49-year-old age group had the most significant incidence of cases, with deaths and disability-adjusted life years (DALY) shifting from children to this age group as well. Pregnancies occur in this age group, and with the incidence being higher in women, it is crucial to have a clear understanding of the physiological changes in pregnancy and the natural progression of dengue to appreciate the potential complications of dengue in pregnancy.

There is also evidence to suggest a higher percentage of severe dengue infections happening to pregnant women than non-pregnant women. Many pregnant women with dengue infection may progress to have Dengue Shock Syndrome (DSS), and the mortality rate triples in this scenario [5].

Dengue has undergone a change in classification by the World Health Organization (WHO). In 1997, symptomatic dengue was divided into undifferentiated fever, dengue fever (DF), and Dengue Hemorrhagic Fever (DHF). DHF was subclassified by severity into four grades. Grades III and IV were known as DSS. The diagnosis of DHF requires a person to have a fever, hemorrhagic manifestation, thrombocytopenia, and evidence of plasma leakage [6].

In addition to the signs seen in DHF, patients who develop DSS additionally have signs of shock, hypotension, a further narrowing of pulse pressure, and an increase in hematocrit (HCT) levels [7,8]. The difficulty in fulfilling the strict criteria of DHF led to the introduction of new criteria in 2009.

In the new criteria, symptomatic dengue was classified as dengue without warning signs, dengue with warning signs (DWS), and severe dengue (SD). Warning signs include abdominal pain and tenderness, persistent vomiting, clinical fluid accumulation, mucosal bleeding, lethargy, restlessness, liver enlargement of more than 2 cm, or laboratory findings of increased HCT value concurrent with a rapid decrease in platelet count. SD is diagnosed when severe plasma leakage leading to shock or respiratory distress, severe bleeding, or severe organ involvement is present [9].

The 2009 WHO classification has less emphasis on pathophysiology and is more like a case management tool. This posed difficulties for dengue research, and the DHF classification continued to be used by researchers [10]. Due to limited research papers following the new classification, this paper includes references to the older classification system.

Despite the high prevalence of dengue in SEA and potentially severe clinical implications in pregnancy, no SEA-specific review is available. Due to varying population genetics, we endeavored to determine the effects of dengue on pregnancy, specifically in SEA, the management options, and the differences in approaches between SEA countries, and further highlight crucial areas for research.

In this review, we discuss the incidence of dengue in SEA, systems-based physiological changes of dengue in pregnancy by trimester, maternal–fetal complications, and relevant investigations and treatments. We aim to provide a concise, up-to-date resource on dengue in pregnancy for healthcare professionals. Simultaneously, we highlight significant gaps in the literature for future research focus.

At the time of writing, we found limited research available to extract data from almost all review sections. Namely, those on the incidence of dengue in pregnancy in SEA, trimester-specific and general pathophysiological changes in dengue, the use of aspirin, preconception vaccination, and impact on the fetus demand a significant proportion of future research.

## 2. Materials and Methods

Our search strategy involved using Google Scholar, PubMed, and Ovid Medline. The latter two were utilized through searching with the following combinations of MESH keywords: “(dengue OR dengue virus) AND (pregnant OR pregnant women) AND (Southeast Asia).” We searched by each country in the geographical SEA (Brunei, Cambodia, Indonesia, Laos, Malaysia, Myanmar, Philippines, Singapore, Thailand, Timor-Leste, and Vietnam). This was used with other relevant keywords using the “OR” conjunction: dengue hemorrhagic fever, dengue shock syndrome, trimester, incidence, investigations, management, monitoring, complications, vaccination, post-partum, fetal/fetal impacts, pre-eclampsia, gestational thrombocytopenia, and gestational diabetes. For two sections, pathophysiology (Section 4) and effect on fetus (Section 5), the data reviewed were not specific to SEA due to the lack of availability. For these sections, the location of origin is disclosed at the start of each section. For the rest of the areas (Management and Preconception Vaccination), data from SEA were primarily targeted for utilization in this review. Due to limited studies based in SEA, we also included combinations of keywords without the “(Southeast Asia)” component. Despite broadening our search through this method, the search yield was limited. As such, the major component of our search strategy included hand-searching based on articles we had previously sourced.

Our inclusion criteria for journal articles included peer-reviewed papers in English, systematic reviews, case–control trials, retrospective studies, cohort studies, and case reports. However, due to a lack of literature from the SEA region, information was sourced from countries outside the SEA. Other references include textbook chapters, national clinical practice guidelines, governmental reports, organization reports, United Nations public health reports, and news reports pertaining to epidemiology and physiology. The data presented in the review will state where they are extracted from for relevant areas. The exclusion criteria were non-English articles and articles without full-text access.

We utilized EndNote x9.3.3 (Clarivate, London, UK) to organize our references.

## 3. Incidence of Dengue in Southeast Asia

There were 5.6 million cases of dengue reported in 2019 globally [3]. This was an 8-fold increase from 2000, when only 505,430 cases were reported. Deaths also increased from 960 in 2000 to 4032 in 2015 [1]. Although 70% of dengue cases are from Asia, human factors such as travel have increased the spread risk in other regions [11].

**Figure 1:**
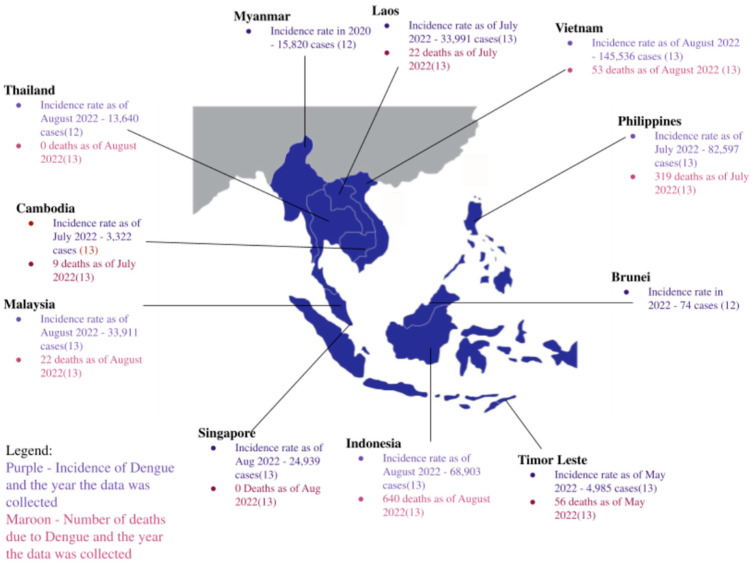
Map of geographical Southeast Asia. The incidence of dengue in each country, the year the data were collected, and the number of mortalities due to dengue [12,13,14].

In SEA, 7,700,000 cases of dengue were reported in 2019. This is a 36.5% increase from 1990, with 4,890,000 cases [4]. Trends and incidences of each country are summarized in Figure 1. Nonetheless, there are no available data looking at the incidence of pregnant women with dengue in SEA.

## 4. Pathophysiology of Uninfected versus Infected Pregnant Women

The first trimester is defined as the period from preconception to 12 weeks, the second trimester of pregnancy from the 13th to the 26th week of pregnancy, and the third trimester from the 27th to the 40th week of pregnancy [15]. Post-partum is defined as the period after the delivery of the fetus. The time frame is not well-specified, but it is generally considered to end around 6 to 8 weeks post-birth [16].

The following tables summarize systemic pathophysiological changes by trimester in pregnancy. Namely, the cardiovascular, endocrinological, hematological, respiratory, and hepatobiliary systems will be discussed. Following each table, further explanation will be given in prose. Physiological changes in thermoregulation will also be addressed.

Careful consideration should be given that the health outcomes measured in the studies referenced may have been complicated by age differences, pre-existing morbidity, different Body Mass Indexes (BMI), healthcare technological advancement variation, or environmental/external factors. Due to varying epigenetics, we attempted to target our search by looking for the pathophysiological changes specific to women in the geographical SEA region. However, due to the lack of research available, we have widened our search to include other demographic data. Such data will be acknowledged in the discussion following every table.

### 4.1. Cardiovascular Physiology

The cardiovascular changes in infected and uninfected mothers are described in Table 1. 

The literature for this section was taken from various countries. We heavily referenced the Sri Lankan guidelines for dengue in pregnancy [7]. The guideline was created using data sourced locally, from SEA, and international guidelines (WHO and Up-to-Date). Of note, it lacked in-text citations to distinguish from where each data point was taken.

Additionally, an older classification system was used to classify dengue according to DHF and DSS rather than newer classifications written by WHO. A SEA guideline on dengue from Malaysia [5] is also referenced in this section. As it is not specific to pregnancy, there is limited information on cardiovascular changes in pregnant women with dengue. For the other articles, one from SEA includes a case report from Singapore [26]. Research based in Asia consists of that from China [20] only. Research based on populations outside of Asia was from Mexico [17], Spain [22], and the United Kingdom [24]. Articles [19,21,23,25,27,28] did not consider or stratify by country of origin.

Decreasing systemic vascular resistance during pregnancy results in a disproportionate fall in diastolic compared to systolic pressures, thus widening the pulse pressure to around 30 mm Hg. During shock, compensatory mechanisms reduce pulse pressures (7). With a fall in oncotic pressures and pulmonary resistance, mothers are also vulnerable to developing APO with any fluid overload or increased capillary permeability [7,27].

Plasma volume decreases during hemorrhage. Due to cardiovascular changes during pregnancy [18], signs of hypovolemia may not display until the later stages, endangering both the mother and fetus [7]. Attending doctors should be aware of this possibility.

Severe dengue increases the risk of pre-eclampsia. Placental ischemia in trimester one can occur secondary to capillary leak syndrome. As inadequate spiral artery development leads to pre-eclampsia, concerns about pre-eclampsia development in the second trimester may arise. With dengue, plasma leakage results from inflammatory cytokines, dengue protein non-structural protein-1 (NS1), and inflammatory lipid mediators, increasing capillary permeability [28]. Maternal compensatory mechanisms following pathological plasma leakage and vasodilation may increase the risk of developing pre-eclampsia [26], especially if dengue was severe (15.4%). In non-severe dengue, pre-eclampsia rates were comparable to that of a normal pregnancy (2.9%). This could be due to the disruption of spiral artery development, although the study does not mention whether the illness onset was in the first trimester, so other mechanisms are possible. In addition, the sample size of this study was small, so further research on a larger scale should be considered. If the positive association between dengue and pre-eclampsia is further confirmed, there may be a need for monitoring infected mothers and prescribing aspirin [17].

Overall, there is limited evidence of cardiovascular physiological changes in infected mothers. Available evidence notes that in infected mothers, there could be a narrowing of pulse pressure [7], increased capillary permeability [7], increased risk of pre-eclampsia [17], and decreased plasma volume [18] in some instances. However, the specific mechanisms of why some of these changes happen are not further explained. Furthermore, the data is not trimester-specific or particular to the SEA population. More research can be done focusing on trimester-specific cardiovascular physiological changes in pregnant women infected with dengue from the SEA region, especially regarding changes to maternal heart rate, maternal BP, and the mechanism behind the changes.

### 4.2. Endocrinological Physiology

The endocrinological changes in infected and uninfected mothers are described in Table 2.

The literature for this section was sourced from various countries. From Asia, one article took data from Asian countries broadly [46], while others used data from India [43], Sri Lanka [47], and Taiwan [36,48]. The literature on populations outside Asia was based on people from Poland [38], the USA [33,42], Denmark [37], and Brazil [49,50,51]. Articles [29,30,31,32,34,35,39,40,41,44,45,52,53] did not consider or stratify by country of origin. No study was based on SEA populations alone.

Placental ischemia in the first trimester can occur secondary to capillary leak syndrome. The resulting placental function dysregulation can alter hPL production. Concerns about developing GDM in the second trimester may arise subsequently as hPL levels are interlinked with GDM. GDM occurs in about 11.5% of pregnancies in Asia [46]. Currently, there is inadequate research into changes in placental hPL production levels. Nevertheless, extraplacental effects of dengue have been shown to cause pancreatomegaly, hyperlipemia, and rarely pancreatitis [43] with an unclear pathophysiology. Postulations include direct virulence on the pancreas, ischemia from DSS, secondary autoimmune reactions, or extraluminal edematous obstruction of the ampulla of Vater [43]. However, there is very little evidence for dengue-induced pancreatitis leading to diabetes mellitus [52] or altering hPL levels during pregnancy.

Progression to the critical phase is more likely to occur in females, diabetics, hypertensives, kidney disease, and those with cardiovascular disease [53]. Studies have shown that pregnancy is also a risk factor for progression to DHF/DSS [49]. A case series from Sri Lanka showed that having GDM was associated with more severe infections such as DHF [47]. This is consistent with studies demonstrating increased severity of illness in Type 2 Diabetes Mellitus patients [48], especially those with poor glycemic control [50]. Dengue also aggravates damage in the diabetic pancreas, with evidence of macrophage infiltrates [51,53]. As such, closer monitoring of at-risk individuals can significantly benefit treatment efficacy, although further studies should confirm this.

To summarize, little evidence can be found on endocrinological changes in infected mothers, especially regarding specific endocrine hormones. More research can be done surrounding trimester-specific endocrinology physiological changes in dengue-infected mothers from the SEA region, specifically looking at the possible changes to hormone levels and the impact of dengue on GDM.

### 4.3. Hematological Physiology

Hematological changes in infected and uninfected mothers are described in Table 3.

The literature for this section was sourced from various countries. From SEA, an Indonesian article [55] and the Malaysia guideline on dengue [5] were used. From Asia, articles used include the Sri Lankan clinical guidelines [7] and research from China if the population discussed was of Chinese ancestry [20,62,64]. Populations outside Asia that were used include England [66]. Articles [7,19,54,56,57,58,59,60,61,63,65] did not consider or stratify by country of origin. There were no studies found based on SEA exclusively.

Research on pregnant Han Chinese women reported them to be in a hypercoagulable state. Han Chinese is the largest Chinese ethnic group in SEA [64,65]. Therefore, physiological changes in Han Chinese and SEA women may have similarities. This data is also consistent with other sources that state that women are in a hypercoagulable state in the second trimester [20].

Thrombocytopenia occurs due to hematopoietic stem cell inhibition and disruption of plasma kinin systems (non-trimester specific). Consumptive coagulopathy can result due to Disseminated Intravascular Coagulopathy (DIC) in severe dengue [63], and prothrombotic states worsen this in pregnancy. Although uncommon, should bleeding occur, early intervention (while taking caution about fluid overload) is crucial for maternal wellbeing [5].

In essence, there is limited evidence of hematological changes in infected mothers. Current evidence suggests that in dengue-infected mothers, thrombocytopenia can occur [63], and a rising HCT can indicate maternal shock [7]. However, leukopenia might not be present in mothers with dengue [7]. The data found are also not trimester-specific and do not look specifically at the physiological changes in infected SEA mothers [7]. Additional research can be done on trimester-specific hematological changes in pregnant women with dengue from the SEA region, looking particularly at how dengue can affect specific blood components, dengue’s impact on coagulability state, and how dengue could further impact mothers with GT.

### 4.4. Respiratory Physiology

Respiratory changes in infected and uninfected mothers are described in Table 4.

The literature for this section was sourced from various countries. From Asia, the Sri Lankan guideline was the only Asian guideline found to be useful [7]. Articles [19,67,69,70] did not consider or stratify by country of origin. No studies were found based exclusively on SEA.

Overall, evidence for respiratory changes in infected mothers is lacking. Available evidence states that infected mothers are more vulnerable to developing APO [7], and respiratory rates might increase [7]. However, there is no evidence on how dengue affects other respiratory parameters, such as the mean partial pressure of carbon dioxide. Data found are also neither trimester-specific nor specific to the SEA population. Further research is needed to examine the trimester-specific respiratory changes in pregnant dengue-infected mothers from the SEA region.

### 4.5. Hepatobiliary Physiology

Hepatobiliary changes in infected and uninfected mothers are described in Table 5.

The literature for this section was sourced from various countries. Articles from populations outside Asia were taken from Mexico [17] and England [72]. Articles [71] and [73] did not consider or stratify by country of origin. There were no studies found based on SEA exclusively.

Dengue can cause maternal liver enzyme derangement. Alanine aminotransferase (ALT) and aspartate transaminase (AST) become elevated [17]. When dengue targets hepatocytes and Kupffer cells, the resultant cell death causes ALT and AST to rise. T-cell-mediated cytotoxicity is precipitated and a brewing cytokine storm of IL-2, IL-6, TNF-α, and IFN-γ (amongst other cytokines), further exacerbates hepatic damage [73].

In summary, some evidence suggests that dengue affects hepatobiliary physiology. Dengue can cause derangement of ALT and AST liver enzymes [17]. However, evidence is lacking on whether ALP, albumin, bilirubin and GGT levels are affected. Furthermore, the data is neither trimester-specific nor SEA population specific. More research is needed on trimester-specific hepatobiliary physiological changes in dengue-infected pregnant women from the SEA region, including looking specifically at changes to the ALP, albumin, bilirubin, and GGT.

### 4.6. Thermoregulatory Physiology

Thermoregulatory changes in infected and uninfected mothers are described in Table 6.

The literature was sourced from various countries. Articles using populations outside Asia include data from the United Kingdom [68] and Norway [74]. Articles [1,19] did not consider or stratify by country of origin. There were no studies found based on SEA exclusively.

The relatively significant increase in estradiol and progesterone in the first trimester, followed by higher estradiol and low progesterone, explains the temperature change. Estradiol causes peripheral vasodilation, which in turn dissipates heat [74].

In essence, the evidence on thermoregulatory physiological changes in infected mothers is neither trimester-specific nor specific to SEA.

### 4.7. Others

The Malaysia guidelines for pregnancy also note an increased risk of dengue encephalopathy in severe dengue. It is not specific to any trimester of pregnancy [5]. The pathogenesis behind this complication includes hepatic encephalopathy, cerebral hypoperfusion in DSS, increased vascular permeability causing cerebral edema, electrolyte imbalance, and intracranial bleeding, which leads to thrombocytopenia or coagulopathy. However, the exact reason why pregnant women have an increased risk of dengue encephalopathy is still unanswered, with researchers questioning whether it is due to the immune-tolerant state of pregnancy [75].

## 5. Impact on Fetus

### 5.1. Vertical Transmission

Although vertical transmission of dengue can happen [5,76], it is rare [77], especially if mothers are asymptomatic [5]. However, a study involving 54 participants in French Guiana reported vertical transmission in about 18.5–22.7%. Dengue is detected by IgM or viruses in the placental, cord or peripheral blood of the newborn. The rate of transmission is higher in the third trimester [78]. Perinatally, dengue is transmitted through the placenta [79]. New evidence suggest that the virus may also be transmitted by breast milk [80]. Clinical presentations of the vertical transmission of dengue in neonates can range from mild (fever) to severe (DHF, DSS, death), where symptoms of fever and rash are most common, followed by hepatomegaly, thrombocytopenia, and DHF.

In a Malaysian study, 63 pregnant women were dengue-specific IgM positive with a vertical transmission rate of 1.6%. Preterm birth rates, low birth weight, and adverse neonatal outcomes were no different from IgM-negative women [81].

The literature for this section was sourced from various countries. From Asia, data from Sri Lanka [77] and Malaysia [5,81] was used. Articles using populations outside Asia used data from French Guiana [82], West Africa [78], Brazil [79], and Polynesia [80]. Article [76] did not consider or stratify by country of origin.

### 5.2. Fetal Malformation

There is no link between dengue with fetal malformations [5,17]. During the first trimester, vertical transmission may raise concerns about defects in organogenesis. Early studies on dengue have concluded that dengue does not cause birth defects [83] or abortions [84]. In addition, no long-term complications are implicated, with the normal development of infants [83]. Another research article also notes that fever during pregnancy adversely affects offspring health, with a 1.5- to nearly three-fold increased risk of developing neural tube defects, congenital heart defects, and oral clefts with maternal fever in the first trimester. Although not dose-dependent, there was some evidence that using antipyretic medications during febrile episodes may have protective effects. Nonetheless, congenital abnormalities in the offspring of women who had dengue in the first trimester may be caused by pyrexia and not be virus-related [85].

The research here was sourced from SEA regions such as Thailand [83], Asian countries such as China [84], and countries outside Asia such as Mexico [17].

### 5.3. Neurodevelopmental Disorder

The risk of neurodevelopmental disorder (NDD) in offspring exposed to maternal fever during pregnancy is increased with an Odds Ratio (OR) of 1.24 [95% CI: 1.12–1.38]. Further, if fever occurred during the first trimester, the risk for NDD increased with an OR of 1.13 [95% CI:1.02–1.26] [86].

### 5.4. Fetal Growth Restriction

Placental ischemia can affect fetal growth. A descriptive Brazilian study proposes that due to capillary leak syndrome and capillary permeability, inadequate vascular supply to the fetus leads to hypoxic injury (loss of trophoblastic epithelia, edema in villous stroma, chorangiosis, infarction), in addition to inflammatory damage (villitis, deciduitis, choriodeciduitis) [87].

Low birth weight is the most common negative impact on the fetus with dengue during pregnancy, according to a French Guiana study [88]. Brazilian data showed DHF doubles the risk of low birth weight, while mild disease increases the risk by 20%. A Thailand study and the Malaysian guidelines on dengue corroborated the higher chances of low birth weight [5,83]. However, other studies showed that dengue does not increase the risk of intrauterine growth restriction (IUGR) [84]. It can be inferred that hypoxia is sufficient to reduce growth rates, but insufficient to lead to IUGR.

### 5.5. Stillbirth

There are a few reported cases of dengue causing fetal death after vertical virus transmission [89]. The study done in French Guiana suggested that the risk of stillbirth increases when the mother has symptomatic dengue infection [88], while another in Thailand showed a higher risk of stillbirth [83]. The Malaysian Guidelines also note the relationship between dengue and fetal death [5].

After 20 weeks of gestation, monitoring for fetal distress can be helpful. In addition to determining fetal status, it may be the first indication of maternal plasma leakage [7]. Fetal tachycardia is observed with fetal hypoxia. Overall, it also stands out that there is a lack of trimester-specific fetal impact, as most research focused on fetal outcomes cumulatively for all trimesters.

### 5.6. Fetal Distress, Delivery, and Maternal Mortality

In an Indonesian study of 41 participants with confirmed dengue during pregnancy, women with secondary infections had more adverse maternal and neonatal outcomes. This included five maternal deaths [90].

Another study done in Mexico suggested that severe dengue increases the risk of fetal distress, cesarean delivery, and maternal mortality in the third trimester of pregnancy [17]. The Thailand guideline that looks at the effect of dengue infection in pregnancy also concurs with the above findings [91].

However, a Thailand study compared 48 pregnant women with dengue to 500 pregnant women without dengue. It concluded that there was no increased risk of adverse neonatal outcomes or maternal mortality [92].

According to the Malaysian guidelines, pregnant women who have spontaneous vaginal delivery while having dengue do not have an increased risk of adverse outcomes. However, infection during labor increases the chances of needing surgical intervention. If that was the case, surgical interventions such as cesarean section and assisted vaginal delivery would increase the risk of bleeding. An infection during labor would also increase the risk of fetal distress [5].

This discrepancy in outcomes shows that more research is needed before a more definitive conclusion can be made on whether dengue affects neonatal and maternal outcomes.

### 5.7. Miscarriage and Preterm Delivery

In Laos, 76 women with confirmed dengue were studied. Six had miscarriages, and nine had preterm births. One participant with severe dengue died [93]. In the Indonesian study of 41 participants mentioned above, one progressed to term, while the other had a miscarriage. Seven women delivered prematurely [90].

In another descriptive study in Brazil, alarmingly, all patients with dengue in the first trimester had miscarriages, possibly due to inflammatory placental changes, as mentioned earlier [87]. This is concurred by the Malaysian guidelines, which state that first-trimester infections are associated with miscarriages [5].

In the Thailand study described above, there was a two-fold increased risk of preterm labor. This indicates a possible link between dengue and miscarriage/preterm delivery [92]. The Malaysian guidelines also note the association between infections in the third trimester and preterm birth [5]. At the same time, the Thailand guidelines state a possible link between dengue and premature uterine contractions [91].

DHF increases the risk of preterm delivery by 10%, according to Thai and Mexican studies [83,94]. This is especially so if the patient is in the critical phase of DHF. If possible, the delivery should be delayed using tocolytic drugs until the plasma leakage is resolved [7]. Steroids usually used to speed up fetal lung development in preterm delivery [95] can still be used. However, there is insufficient evidence to prove the use of steroids in the treatment of dengue [96].

Sources [95] and [96] did not consider or stratify by country of origin.

### 5.8. Placental Abruption and Hemorrhage

Placental abruptions can happen any time after 20 weeks of pregnancy [97]. Indonesia reported the only placental abruption occurring at 35 weeks of gestation due to DHF [98].

According to the Thailand guidelines, dengue can increase the risk of intrapartum and post-partum hemorrhage [91]. Medical teams should consider how this could impact the management of pregnancy and delivery of infected pregnant women.

## 6. Management

Despite the severe implications of dengue in pregnancy and its increasing prevalence in SEA, data on its management in SEA are scarce, restricting any discourse. There are five guidelines discussing the management of dengue from SEA. They are from Malaysia [5], Singapore [99], the Philippines [100], Myanmar [101], and Thailand [91].

### 6.1. Investigations

In SEA, guidelines on dengue are available from Myanmar [101], the Philippines [100], Thailand [91], Malaysia [5], and Singapore [99]. Investigations recommended by the five countries vary slightly. All guidelines described the general investigations done for adults with dengue. Only the Myanmar [101] and the Philippines [100] guidelines outlined the investigations essential to pregnant individuals. The guidelines from the Philippines [100] and Singapore [99] lacked information on recommended investigations for follow-up. Thailand’s [91] guidelines were brief and failed to mention the initial and follow-up tests that should be done. The dengue guidelines from Brunei, Indonesia, Laos, Timor-Leste, and Vietnam were unavailable, while the Cambodian guidelines were inaccessible. Therefore, in this section, we consolidated the available guidelines in Table 7. Information in the table is applicable for dengue in adults unless specified. 

The collated information in Table 7 shows that the investigations recommended are relatively similar. Where an initial assessment was mentioned in the guidelines, an FBC including HCT is recommended, and dengue IgM/IgG tests are also generally listed as a diagnostic tool.

Nonetheless, several guidelines still have gaps in the investigation section. This is especially so in the required follow-up investigations. It is essential to highlight that the guidelines were mostly investigations for adults suspected of having dengue and not pregnancy specific. Myanmar [101] and the Philippines [100] are the only two countries that mentioned investigations that should be done on pregnant women. Myanmar’s guidelines stated that FBC needs to be repeated daily once the pregnant woman is admitted [101], while the Philippines guidelines said that FBC is essential in a pregnant woman [100]. These guidelines, however, do not adequately address the need for additional pregnancy specific investigations, like a biophysical profile or cardiotocography, to assess fetal wellbeing.

Regarding the initial assessment, all guidelines apart from Thailand [91] stated FBC, including HCT, as their investigation of choice. Myanmar [101], Malaysia [5], and Singapore [99] guidelines also mentioned other baseline investigations. The Philippines was the only country that specified history and examinations that should be done to help rule in/out the diagnosis of dengue [100]. Thailand’s guidelines failed to mention the initial investigations that should be carried out [91]. While FBC, including HCT, may be adequate in a general adult, medical teams should consider the need for a further initial assessment in pregnant individuals.

All five guidelines mentioned the investigations used for diagnosing dengue. However, as previously mentioned, they are not pregnancy specific and vary slightly between countries. Dengue PCR is the only investigation recommended by all five countries. There are also differences in opinions on when the dengue IgG test should be done, with Myanmar recommending it from day 5 [101], while Malaysia recommends it from day 7 [5]. Although test sensitivity in the different labs might be a reason for the difference, further exploration to determine the day might be helpful. Diagnostic tools for dengue are available, but one specific for dengue in pregnant women is unavailable.

Follow-up investigations that are required for pregnant women with dengue are lacking. Only the Malaysian and Myanmar guidelines mentioned the necessary follow-up investigations, and only FBC was listed [5,101]. All the guidelines require a more in-depth list of follow-up investigations to assist medical teams in managing pregnant women with dengue.

The dengue guidelines available from SEA were not pregnancy specific. Important differential diagnoses such as HELLP (hemolysis, elevated liver enzymes, and thrombocytopenia) syndrome in patients with pre-eclampsia were not discussed.

### 6.2. Treatment

Approaches to dengue in pregnancy vary in the clinical practice guidelines of SEA countries. The guidelines found (Myanmar, Philippines, Malaysia, and Thailand) described general adult management of dengue based on various phases of the disease. Special considerations for pregnant individuals were subsequently described. The guideline from Singapore was brief and did not consider pregnancy status. Similarly, Thailand guidelines had limited information about dengue management and even less about dengue during pregnancy. Some SEA countries had no guidelines available online (Brunei, Indonesia, Laos, Timor-Leste, and Vietnam) or required permission to view (Cambodia). For this section, we will consolidate the guidelines specific to dengue in pregnancy, and a summary table is shown below (Table 8).

Myanmar’s and the Philippines’ guidelines bear similarities. They designated pregnancy as one of the many indications for in-hospital management, then detailed how the latter should be done. As such, although these guidelines contain the highest quantity of relevant data amongst SEA countries, the management was not specific to dengue in pregnancy. Table 8 summarizes which SEA countries considered pregnancy-specific information in their guidelines and provides links to each guideline.

A comparison of guidelines from SEA is described in Table 9.

From Table 9, it can be inferred that the guidelines bear some similarities. Some examples include higher precaution levels, monitoring parameters, transfusion guidelines, and reduction of traumatic procedures peripartum.

However, many of the guidelines require more management information. This is especially so for dengue and its various phases, and dengue in the pregnant state. While some guidelines appear to fill the gaps of other guidelines, most of the data are not pregnancy-specific. As mentioned earlier, the guidelines from Myanmar and the Philippines [100,101] merely stated pregnancy as one of the indications in an overall management flow without differentiating management for DHF, DSS, or progression of complications in pregnancy. For instance, the Philippines guidelines provide extensive detail about fluid management, but do not consider the hypervolemic state in pregnancy [100]. Fluid management not tailored to pregnancy could overcorrect and cause fluid overload, resulting in acute pulmonary edema [7,27]. Pregnancy-specific comorbidities such as pre-eclampsia and gestational diabetes were not considered. Although the Myanmar guidelines did mention lower platelet levels in pregnancy, gestational thrombocytopenia was not addressed. The Myanmar guidelines were also the only ones to describe physiological changes to vital signs in pregnancy.

Additionally, all the guidelines had neither trimester-specific information nor fetal surveillance advice.

Lastly, only the Myanmar and the Philippines guidelines considered the 2009 WHO classification of dengue (DWS, SD) and stratified management based on this. The Myanmar guidelines included DHF and DSS management, while the Philippines guidelines were entirely based on the 2009 classification. As opposed to the Malaysia guidelines, the Myanmar and Philippines guidelines do not mention the management of organ impairment that can occur in SD. The Malaysia, Thailand, and Singapore guidelines outlined management based on older classifications (DHF, DSS).

No guidelines specific to dengue in pregnancy were available in the SEA region. However, Sri Lankan guidelines (South Asia) have extensive details on dengue in pregnancy and can be helpful as a reference [7].

#### Ongoing Use of Aspirin

For the use of aspirin, the literature was taken from various populations. From Asia, the Sri Lankan [7] and Indian guidelines [102] were used. Articles [103,104,105] did not consider or stratify by country of origin.

Patients with a history of pre-eclampsia or antiphospholipid syndrome may already be on prophylactic low-dose aspirin therapy, which without dengue has low risks of bleeding [103]. Low-dose aspirin is an irreversible Cyclooxygenase-1 inhibitor with an antiplatelet effect via thromboxane-A2 [104]. This may potentiate the increased risk of bleeding from dengue.

For those at risk of pre-eclampsia and its complications (such as pulmonary embolism or IUGR), 75–150 mg aspirin is given orally once daily from 12–16 weeks of gestation until delivery [105]. Ongoing use of aspirin can precipitate bleeding [7] and should be withheld if infected with dengue, as it can worsen thrombocytopenia [102]. However, further research is needed to investigate the benefits and costs of using antiplatelet therapy.

Overall, investigations and management depend on the resources the hospital has access to and may differ across geographical locations. However, the principles of management (early suspicion of dengue, early monitoring for complications, managing fluid and aspirin use) remain unchanged. Aside from the Sri Lankan guidelines, there is a lack of available management guidelines and evidence about maternal aspirin use in dengue, suggesting a need for an increased focus on managing dengue in pregnancy, especially in endemic SEA countries.

## 7. Preconception Vaccination

For preconception vaccination, the literature was taken from various populations. From SEA, a Malaysian study [106], a Singaporean study [107], and a Vietnamese study [108] were used. Articles [109,110,111] did not consider or stratify by country of origin.

With pregnant women having poorer outcomes in dengue, is there a role for preconception vaccination?

Dengvaxia^®^ (CYD-TDV), the only licensed dengue vaccine globally, is a live attenuated tetravalent vaccine that can improve immunity against all dengue virus serotypes only for seropositive (70% or higher seroprevalence) individuals in endemic areas.

It is contraindicated in seronegative individuals as it can precipitate poorer outcomes than in the unvaccinated [109]. WHO cautions against Dengvaxia in pregnant or lactating women as there is inadequate research. A small sample size study showed no increase in adverse pregnancy outcomes following the Dengvaxia administration [110]. This finding is consistent with a Malaysian study where seropositive mothers (secondary to infections) tend to transfer IgG cross-placenta without adverse effects on fetuses [106].

A study on mice based in Singapore found that vaccinated pregnant mice are likely to vertically transfer maternal strain-specific antibodies to their offspring for the first few months post-partum. More significantly, the offspring also developed a strong cross-reactive immunoprotective CD8 T cell response that suppressed viral replication. However, they were still susceptible to Antibody Dependent Enhancement (ADE) of infection with heterologous strain infections, causing severe disease [107]. This occurs in secondary infections when antibody–antigen complexes do not neutralize viruses, but facilitate further adsorption into host leukocytes, precipitating higher viral loads [111]. Further research on Vietnamese infants showed that declining maternal IgG to critical levels can lead to ADE and that DHF was likely only to be present in infants if the mothers are seropositive [108]. Further preconception studies are needed to confirm the fetal impact.

Overall, preconception vaccination is not recommended due to a lack of data. For seronegative individuals, maternal antibodies confer immunity to infants through antibody transfer. However, this is short-term and could cause infant predisposition to ADE. Even though there has been no reported adverse neonatal impact from seropositive individuals, there is still insufficient information on vaccination safety. The idea of seropositive preconception vaccination has yet to be explored by research. As there is limited Asian population-based research on preconception vaccination, further studies must be done to investigate the precise cost–benefit ratios.

## 8. Areas for Improvement

This review highlights specific areas for further research.

For epidemiology, the incidence of dengue in pregnant women in SEA is unknown. Regarding physiology, research on infected pregnant women is lacking for all the systems discussed, especially trimester-specific and SEA-specific information. For the cardiovascular system, the mechanisms of physiological changes in infection and trimester-specific pathological effects on vital signs in infection require further research. Future research should focus on gestational BP due to the higher pre-eclampsia risk in severe dengue, especially in SEA women. For the endocrinological system, no data was available detailing dengue’s impact on various hormone levels. More importantly, there was inadequate evidence on the effect of dengue on GDM risk. For hematology, there is insufficient trimester-specific data about how dengue affects various blood components, the coagulability state, and the effects on GT. This is important, as they provide parameters for measuring the baseline and scoring severity. Specifically, the effects on GT are crucial due to implications on hemorrhagic risks. Pertaining to hepatobiliary physiology, there is a lack of evidence on changes to ALP, albumin, bilirubin, and GGT levels. In terms of respiratory physiology, more research is needed to determine how dengue affects respiratory parameters, such as the mean partial pressure of carbon dioxide, for baseline and severity scoring.

In terms of management, some SEA guidelines did not consider pregnancy status extensively, if at all. None of the SEA guidelines were wholly pregnancy-specific. Although the various guidelines fill each other’s gaps, cross referencing may not be applicable due to variability in consideration of pregnancy status. The management of pregnancy complications in dengue-infected mothers, such as pre-eclampsia, gestational thrombocytopenia, or gestational diabetes, was not explicitly covered. SEA guidelines that were utilized either did not stratify management by the 2009 WHO guidelines’ classification of dengue (DWS, SD) or had incomplete information (failure to explain appropriate care in SD with organ failure). Additionally, insufficient research into the outcomes and indications for aspirin cessation in pregnant mothers with pre-eclampsia risks restricts management guidelines.

Moreover, preconception vaccination’s maternal and fetal outcomes, especially for pregnant seropositive women in SEA, require further research as no data are available. Regarding fetal complications, there is a lack of trimester-specific effects of DHF and DSS (most articles displayed fetal outcomes cumulatively for all trimesters). Additionally, trimester-specific effects on fetal platelet counts need further research.

Further, insufficient information is available to guide the management of aspirin use for pre-eclampsia in the setting of the hemorrhagic risk of dengue. While the current treatment method is aspirin cessation, perhaps titration to lower doses can achieve optimal therapeutic benefits for the patient. There is also inadequate research on preconception vaccination available to conclude the vaccination benefits versus costs for seropositive and seronegative pregnant women, much less SEA-based conclusions. There is a need to investigate the protective effects versus the ADE risk for vaccination of mothers.

Lastly, larger sample size studies are needed to reduce sampling errors.

## 9. Conclusions

The physiological cardiovascular, respiratory, hematological, and endocrine changes in an infected pregnant woman that are necessary for an uncomplicated pregnancy and birth can be altered, leading to complications such as placental abruption, preterm delivery, low birth weight, and stillbirth. Investigation and management of dengue fever in pregnant women are mostly like those of non-pregnant individuals. Management requires early suspicion of dengue, serology tests, managing fluid status and hemodynamic status, and preparing for progression to critical phases. Without adequate management, DHF or DSS can lead to bleeding, multi-organ failure, fetal distress, and predisposal to miscarriages, especially in the first trimester.

The fetus can be adversely affected through direct vertical transmission or secondary to complications of maternal infection. Although dengue in the fetus does not cause long-term complications, monitoring vertical transmission via infant serum serology is recommended. Therefore, the monitoring of fetal wellbeing in pregnant women with dengue should be considered.

In general, there is a lack of research on dengue in pregnancy, especially in SEA. Thus, reviewing investigation and management guidelines for dengue-infected women in different stages of pregnancy has significant restrictions. We recommend a substantial increase in research on the areas listed above. More data on various aspects of dengue in pregnancy, such as pathophysiological changes, response to the disease processes, and management in SEA women, needs to be explored.

Our review had some limitations. The most relevant limitation is the unavailability of literature based on SEA, necessitating the utility of data from other populations. The literature available also did not utilize WHO guidelines classification of dengue into DWS and SD. Hence, this review was based on the older classification of dengue. Additionally, the Sri Lankan study referenced in this review did not have in-text citations to support their data points, and their bibliography included data from non-native populations.

## Figures and Tables

**Table 1:** Uninfected versus dengue-infected cardiovascular changes in pregnant women.

Gestational Stage	Uninfected	Infected
Trimester 1	Widened pulse pressure [7].	Higher risk of pre-eclampsia and eclampsia in severe dengue. Unclear if related to first or second-trimester infection [5,17]. Narrowing of pulse pressure to 25 mm Hg or less with shock [7]. Sepsis or DHF increases capillary permeability, putting infected mothers at an even higher risk of Acute Pulmonary Edema (APO) [7]. Decrease in plasma volume can happen secondary to bleeding in dengue [18].
Trimester 2	Development of pre-eclampsia in susceptible women. Diastolic BP drops more than systolic BP, causing widened pulse pressure [7] Mean heart rate = 75 beats per minute (bpm) [19]. Blood pressure (BP) generally reduces [20]. Progressive increase in plasma volume [21].	Effects on pre-eclampsia and pulse pressures are as above. Refer to above for general altered physiology for dengue in pregnant women.
Trimester 3	The mean heart rate peaks late in the third trimester. However, it should not be >95 bpm [7]. Plasma volume increases until the 30th–34th week and then plateaus or decreases slightly through the term [21]. BP generally increases [22]	No specific altered physiology for dengue in pregnant women in the third trimester can be found. Refer to above for general altered physiology for dengue in pregnant women.
Post-partum	Two weeks post-delivery, the mother returns to a hemodynamically same state as pre-pregnancy [23]. Maternal heart rate is fastest on the day of delivery, with a median of 83 bpm. This decreases until Day 14 post-labor [24]. Maternal BP increases until day 5 or 6 post-delivery, reaching a median of 121/79 mm Hg. It then decreases to normal by day 14 post-partum, reaching a median of 116/75 mm Hg [24]. Plasma volume decreases [25].	No specific altered physiology for dengue in post-partum women can be found.

**Table 2:** Uninfected versus dengue-infected endocrinological changes in pregnant women.

Gestational Stage	Uninfected	Infected
Trimester 1	Progesterone level increases [29]. Human chorionic gonadotropin (hCG) peaks from the 8th to the 10th week before plateauing [30]. Relaxin increases [30]. Parathyroid hormone levels rise [31]. Prolactin levels rise [32].	There is insufficient research on pathological hormonal changes in infected pregnant mothers.
Trimester 2	GDM can develop in susceptible women. Increase in human Placental Lactogen (hPL) levels [33] stimulates liver production of insulin-like growth factor (IGF-1) [34] for glucose control [35]. High levels of IGF-1 have an insulin-like effect [36]. However, extremely high levels are linked to insulin resistance and could increase the risk of developing Type 2 Diabetes Mellitus [37]. Human leukocyte antigen-G (HLA-G) increases to regulate the immunological system [38]. Reduced serum HLA-G increases the risk of pre-eclampsia [39,40,41]. Increase in estrogen levels [42]. Causes vasodilation of blood vessels, thus increasing blood flow to the uterus and placenta [30].	Lack of evidence to suggest dengue increases the risk of developing GDM [43]. No evidence was found on how dengue can affect the hormones discussed.
Trimester 3	hPL levels do not change much from the 2nd trimester [33]. Serum HLA-G levels decrease in concentration (63.31 Units/mL), but remain higher than in non-pregnant women [38]. Estrogen continues to increase [44].	No specific altered physiology for dengue in pregnant women in the third trimester can be found. Refer to above for general altered physiology for dengue in pregnant women.
Post-partum	hPL levels decrease post-birth, becoming undetectable by 18–24 h post-birth [33]. Estrogen levels decrease after placenta removal and return to pre-pregnancy levels by day 5 post-partum [45].	No specific altered physiology for dengue in post-partum women can be found.

**Table 3:** Uninfected versus dengue-infected hematological changes in pregnant women.

Gestational Stage	Uninfected	Infected
Trimester 1	Lymphocyte count decreases, although leukocytosis is present [54]. Blood volume expansion at 6–12 weeks [54]. Fall in hemoglobin (rise in hemoglobin is outweighed by the increase in plasma volume) [54].	Leukopenia may not be seen in infected mothers [7]. Due to physiological hemodilution during pregnancy, hemoconcentration with plasma leakage in dengue may be masked [55]. Therefore, a rising HCT can mean maternal shock [7].
Trimester 2	Increased hypercoagulability, particularly in the second trimester [20]. Driven by an elevation of factors VII, VIII, X, von Willebrand factor, and fibrinogen. Reduced sensitivity to activated protein C and protein S [56] and increased inhibition of fibrinolytic factors [57]. Mechanical factors like increased abdominal pressure on pelvic veins favor hemostasis as the pregnancy progresses [57]. Estrogen is also prothrombotic and increases the risk of venous thrombosis [58]. Increased platelet count [20]. Gestational thrombocytopenia (GT) may develop in 4.4% to 11.6% of pregnant women [59]. GT happens as platelet counts fall below 130–150.000/ìL [60]. It rarely drops <80 × 10^9^/L in GT [7]. Hemodilution from higher plasma volume, placental consumption of platelets [61], and possible inefficacious thrombopoietin action [62] result in GT. Usually asymptomatic, with no significant consequences from gestational thrombocytopenia [61]. Mean white blood cell (WBC) is around 8 × 10^9^/L [19], increased [20]. Decreased erythrocyte count [20].	Thrombocytopenia can occur in dengue [63], and a fall in platelet counts below <80 × 10^9^/L may suggest plasma leakage [7].
Trimester 3	Mean WBC count approximates 9 × 10^9^/L [19]. Leukocyte count remains like the second trimester [20]. Platelet count decreases [20]. Erythrocyte count increases from the 28th week to the end of pregnancy [20]. Remains in a hypercoagulable state [64,65].	No specific altered physiology for dengue in pregnant women in the third trimester can be found. Refer to above for general altered physiology for dengue in pregnant women.
Post-partum	Leukocyte count peaks a week post-partum and subsequently decreases drastically at week 7 post-partum [20]. Platelet count increases after birth and peaks a week post-partum [20]. Erythrocyte count decreases a week post-partum before increasing again [20]. Hypercoagulable state takes approximately 4 weeks to resolve [66].	No specific altered physiology for dengue in post-partum women can be found.

**Table 4:** Uninfected versus dengue-infected respiratory changes in pregnant women.

Gestational Stage	Uninfected	Infected
Trimester 1	Minute ventilation increases by up to 48% with higher tidal volume and unchanged respiratory rate [67].	No specific altered physiology for dengue in pregnant women in the first trimester can be found. Refer below for general altered physiology for dengue in pregnant women.
Trimester 2	Pregnancy leads to compensated respiratory alkalosis. Increased oxygen consumption causes hypoxia susceptibility [7]. Mean respiratory rate is around 15 breaths per minute [19]. Mean partial pressure of carbon dioxide is around 32 mm Hg [19].	No trimester-specific evidence on the altered respiratory physiology in pregnant women with dengue was found. The fall in oncotic pressure and pulmonary resistance causes vulnerability to developing APO [7]. Additionally, the respiratory rate might increase [7].
Trimester 3	Continued compensated respiratory alkalosis and oxygen consumption [7]. Respiratory rate ranges between 8–24 breathes per minute [68].	No specific altered physiology for dengue in pregnant women in the third trimester can be found. Refer to above for general altered physiology for dengue in pregnant women.
Post-partum	Respiratory parameters return to normal 6–12 weeks after labor [69]. Respiratory rate returns to pre-pregnancy levels by 2–3 days post-birth [70].	No specific altered physiology for dengue in post-partum women can be found.

**Table 5:** Uninfected versus dengue-infected hepatobiliary changes in pregnant women.

Gestational Stage	Uninfected	Infected
Trimester 1	Albumin level decreases [71]. Serum total and free bilirubin concentrations are lower in all trimesters [71]. Serum alkaline phosphatase (ALP) activity increases [71]. Serum gamma-glutamyl transferase (GGT) activity levels decrease slightly [71].	Maternal liver enzyme derangement [17].
Trimester 2		
Trimester 3		
Post-partum	Bilirubin concentration increases post-partum, returning to pre-pregnancy levels by day 5 post-partum [72]. GGT levels should return to normal by day 13 post-partum [72]. GGT levels initially decrease, then increase, peaking at either day 5 or 10 post-partum [72].	

**Table 6:** Uninfected versus dengue-infected thermoregulatory changes in pregnant women.

Gestational Stage	Uninfected	Infected
Trimester 1	Core body temperature increases a few tenths of a degree [74].	Dengue causes an increase in body temperature [1]. No trimester-specific data was found.
Trimester 2	Mean body temperature decreases from the first trimester [74] to around 36.9 degrees Celsius [19].	
Trimester 3	Body temperature decreases from the second trimester [74] and ranges between 35.37 to 37.35 degrees Celsius [68].	
Post-partum	Core body temperature remains below pre-pregnancy levels until 3 months post-partum [74].	

**Table 7:** Comparison of investigations recommended by dengue guidelines from Myanmar [101], the Philippines [100], Malaysia [5], Thailand [91], and Singapore [99].

Country	Initial Assessment	Diagnostics	Follow-Up
Myanmar	Baseline Full Blood Count (FBC), including HCT In the presence of leukopenia and/or thrombocytopenia and with signs of dengue should be sent for a medical consultation. Changes to baseline parameters should be noted as early as possible If HCT is unavailable, multiply hemoglobin by 3 for an estimate of HCT. Other tests to consider: Blood glucose, serum electrolytes, calcium, urea, creatine, bicarbonate, coagulation profile, liver function tests	Dengue NS1 test Dengue IgM test Recommended from day 5 Highly suggestive of dengue: Positive in a single serum sample: Confirmed dengue: IgM seroconversion in paired sera Dengue IgG test Recommended from day 5 Highly suggestive of dengue: Positive in a single serum sample with hemagglutination inhibition of more than or equal to 1280: Confirmed dengue: IgG seroconversion in paired sera or fourfold IgG titre increase in paired sera Duo test (NS1 Ag + IgM/IgG) Recommended from day 4 As a duo test, the sensitivity may increase to >90% Dengue Polymerase Chain Reaction (PCR) Confirmed: Positive Dengue Virus culture Confirmed: Positive	FBC Monitor for signs of DHF—leukopenia, thrombocytopenia, and a rising HCT FBC Checked daily in pregnant women admitted to the hospital
Philippines	History Physical examination Signs of ascites, tourniquet test, signs of rash and bleedings, hemodynamic status, signs of respiratory distress Investigations: FBC, including HCT (essential in pregnant women)	Viral culture isolation Dengue PCR Diagnostic tests are not necessary for acute management.	Not Applicable (N/A)
Malaysia	Baseline FBC (including HCT) for all patients suspected of having dengue. (As per point 1 of Myanmar guideline) Other tests to consider performing include liver function test, renal profile, coagulation profile, lactate, blood gases, troponin, and creatine kinase.	Rapid Combo Test (RCT) Analyzes the presence or absence of dengue NS1 Antigen, dengue IgM and IgG antibodies NS1 Antigen detection test for NS1 antigen is necessary for virus viability. Dengue IgM test IgM enzyme-linked immunosorbent assay (ELISA) More helpful in picking up primary infections compared to secondary infections Dengue IgG test IgG ELISA Detected 7 days after onset of Fever Repeat is recommended if dengue IgM is still negative after day 7 and the initial IgG test showed a negative result. Useful in differentiating primary and secondary dengue infections Dengue Viral RNA Detection (RT-PCR) Only useful during the viremic stage Detects viral RNA up to 5 days after symptom onset Detects dengue serotype Dengue Virus Isolation/Culture Only carried out for research, surveillance, and genotyping purposes	Serial FBC to monitor disease progression
Thailand	N/A	Dengue PCR Dengue NS1 Dengue IgM/IgG test	N/A
Singapore	FBC (including HCT). (As per point 1 of Myanmar guideline) Liver Function Tests Monitor for elevated transaminases (AST is normally more elevated than ALT)	Dengue IgM As per the Myanmar guidelines Dengue IgG As per the Myanmar guidelines Dengue PCR To be done within 5 days of onset of symptoms	N/A

**Table 8:** Data from SEA countries with pregnancy-specific information.

	Malaysia [5]	Thailand [91]	Myanmar [101]	Singapore [99]	Philippines [100]
Pathophysiology				N/A	N/A
Investigations					
Management					
Complications				N/A	N/A
Links	https://www.moh.gov.my/moh/resources/penerbitan/GUIDELINE/GUIDELINE%20Dengue%20Infection%20PDF%20Final.pdf	https://www.tm.mahidol.ac.th/seameo/2015-46-1-suppl/c7Annexp169-181.pdf	https://www.mmacentral.org/wp-content/uploads/2022/06/National-Guideline-for-Clinical-Management-of-Dengue-2021.pdf	https://www.ncid.sg/Health-Professionals/Diseases-and-Conditions/Pages/Dengue.aspx	https://pcp.org.ph/images/PSBIM/PSBIM_Local_Guidelines_2019/RevisedDengueClinicalCaseManagementGuidelines2011-DOH.pdf
Access Date	2 December 2021	9 December 2022	9 December 2022	9 December 2022	9 December 2022

Legend: 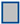 Pregnancy specific data. 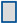 Absence of pregnancy specific data. 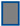 Mixed data (standard dengue management with some evaluation for pregnancy). “N/A” refers to “Not Applicable.” Guidelines from Brunei, Indonesia, Laos, Timor-Leste, Vietnam, and Cambodia were not assessed.

**Table 9:** Comparison of guidelines from Myanmar [100], the Philippines [99], Malaysia [5], Thailand [90] and Singapore [98].

Country	Initial Assessment	Febrile Phase	DHF	DSS	Complications	Peripartum
Myanmar	Admit if Day 2 of fever, with multidisciplinary team involvement. Hemodynamic stability should consider Lower baseline BP Higher pulse pressure Higher heart rates FBC interpretation should consider Lower HCT Lower platelet counts Fluid management Calculated based on preconception weights	N/A	DHF with warning signs: Reference HCT taken Isotonic intravenous (IV) fluids given Vital signs, perfusion status, urine output, and blood glucose or organ function tests are monitored for improvement or deterioration into shock In DHF without warning signs: Oral fluids preferred over IV Same monitoring is done as for DHF with warning signs	Transfer to high dependency or intensive care. Immediate crystalloid fluid resuscitation.	If profound thrombocytopenia Strict bed rest Avoidance of trauma Prophylactic platelet transfusion is not evidence-based Severe hemorrhage 5–10 mL/kg of fresh-packed red blood cells (PRBC) administered as needed, and patient monitoring for improvement	Avoidance of cesarean sections, induction of labor, and obstetric procedures (due to hemorrhagic risk). If necessary, interventional delivery Maintain platelet counts of about 50,000/mm^3^, with potential platelet single donor transfusion Episiotomy to prevent perineal tears Post-partum fever Early suspicion of dengue and physician referral
Philippines	N/A	N/A	As per the Myanmar guidelines. Contains detailed guidelines on fluid management in DHF without warning signs.	Hypotensive shock Extensive guidelines on IV fluid management If HCT uncorrected with fluids, suspect hemorrhage.	As per the Myanmar guidelines.	N/A
Malaysia	Close monitoring of vital signs for shock. Appropriate fluid or blood product administration. Early referral to intensivist and obstetrician.	Measure hemodynamic, respiratory, neurological status, and urine output every 4 h. FBC daily. Organ function tests, Arterial Blood Gas (ABG), lactate, and coagulation profile, only if indicated.	Measure hemodynamic, respiratory, neurological status, and urine output every 2 h. FBC 4–12 hourly. Renal and liver function tests, creatine kinase daily at least. Cardiac function tests, ABG, lactate, and coagulation profile, only if indicated.	Measure hemodynamic, respiratory, and neurological status every 15 min until stable, then hourly. Measure hourly urine output. FBC between fluid resuscitation. ABG, lactate close monitoring. Organ function tests, and coagulation profile, only as indicated.	As per the Myanmar guidelines. Contains detailed management of hepatic, cardiac, neurological, and immunological complications of dengue, and intensive care management.	Avoidance of caesarean section, operative vaginal delivery, intramuscular injections. Blood products, including platelets, on standby if interventional delivery needed. Spontaneous delivery preferred. Preterm labor Tocolysis (nifedipine or atosiban). Close fetal monitoring. Group and cross match. Third stage of labor IV uterotonic agent. Breastfeeding recommended only after the viremic phase.
Thailand	N/A	Monitoring similar to the Malaysian guidelines but does not distinguish between different phases of dengue.			N/A	As per the Myanmar guidelines on intrapartum platelet transfusion.
		Antipyretics, hydration, and supportive care (no clear indication of specific phase involved).	N/A	Persistent shock despite fluids - Determine if other causes are present (including extreme hemorrhage) - IV vasopressors like norepinephrine		
Singapore	N/A	As per the Thailand guidelines on supportive care Daily platelet and HCT for platelets less than 100,000/mm^3^ Complete bed rest for platelets less than 50,000/mm^3^			N/A	N/A

Legend: 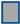 Pregnancy-specific data. 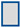 Lack of pregnancy-specific data. 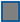 Mixed data (standard dengue management with some evaluation for pregnancy). “N/A” refers to “Not Applicable “.

## Data Availability

Not applicable.

## Abbreviations

ABG	Arterial Blood Gas
ADE	Antibody Dependent Enhancement
ALP	Alkaline Phosphatase
ALT	Alanine Transaminase
APO	Acute Pulmonary Edema
ASIR	Age-Standardized Incidence Rate
AST	Aspartate Aminotransferase
BMI	Body Mass Index
BP	Blood Pressure
Bpm	Beats per minute
CYD-TDV	Dengvaxia
DALY	Disability-Adjusted Life Years
DHF	Dengue Hemorrhagic Fever
DIC	to Disseminated Intravascular Coagulopathy
DSS	Dengue Shock Syndrome
DWS	Dengue with Warning Signs
ELISA	Enzyme-Linked Immunosorbent Assay
FBC	Full Blood Count
GDM	Gestational Diabetes Mellitus
GGT	Gamma-glutamyl Transferase
GT	Gestational Thrombocytopenia
hCG	Human Chorionic Gonadotropin
HCT	Hematocrit
HELLP	Hemolysis, Elevated Liver enzymes, Low Platelets
HLA-G	Human Leukocyte Antigen G
hPL	Human Placental Lactogen
IUGR	Intrauterine Growth Restriction
IV	Intravenous
N/A	Not Applicable
NDD	Neurodevelopmental Disorder
NS1	Non-Structural Protein 1
OR	Odds Ratio
PCR	Polymerase Chain Reaction
PRBC	Packed Red Blood Cells
RCT	Rapid Combo Test
SD	Severe Dengue
SEA	Southeast Asia
WBC	White Blood Cell count
WHO	World Health Organisation
